# IMRT and RapidArc commissioning of a TrueBeam linear accelerator using TG‐119 protocol cases

**DOI:** 10.1120/jacmp.v15i5.4843

**Published:** 2014-09-08

**Authors:** Ning Wen, Bo Zhao, Jinkoo Kim, Karen Chin‐Snyder, Maria Bellon, Carri Glide‐Hurst, Kenneth Barton, Daiquan Chen, Indrin J. Chetty

**Affiliations:** ^1^ Department of Radiation Oncology Henry Ford Health System, Detroit Michigan USA

**Keywords:** commissioning, flattening filter‐free, RapidArc, GAFCHROMIC EBT films, TG‐119

## Abstract

The purpose of this study is to evaluate the overall accuracy of intensity‐modulated radiation therapy (IMRT) and RapidArc delivery using both flattening filter (FF) and flattening filter‐free (FFF) modalities based on test cases developed by AAPM Task Group 119. Institutional confidence limits (CLs) were established as the baseline for patient specific treatment plan quality assurance (QA). The effects of gantry range, gantry speed, leaf speed, dose rate, as well as the capability to capture intentional errors, were evaluated by measuring a series of Picket Fence (PF) tests using the electronic portal imaging device (EPID) and EBT3 films. Both IMRT and RapidArc plans were created in a Solid Water phantom $\left(30\;\times \;30\;\times \;15\;{\text{cm}}^{3}\right)$ for the TG‐119 test cases representative of normal clinical treatment sites for all five photon energies (6X, 10X, 15X, 6X‐FFF, 10X‐FFF) and the Exact IGRT couch was included in the dose calculation. One high‐dose point in the PTV and one low‐dose point in the avoidance structure were measured with an ion chamber in each case for each energy. Similarly, two GAFCHROMIC EBT3 films were placed in the coronal planes to measure planar dose distributions in both high‐ and low‐dose regions. The confidence limit was set to have 95% of the measured data fall within the tolerance. The mean of the absolute dose deviation for variable dose rate and gantry speed during RapidArc delivery was within 0.5% for all energies. The corresponding results for leaf speed tests were all within 0.4%. The combinations of dynamic leaf gap (DLG) and MLC transmission factor were optimized based on the ion chamber measurement results of RapidArc delivery for each energy. The average 95% CLs for the high‐dose point in the PTV were 0.030 ± 0.007 (range, 0.022–0.038) for the IMRT plans and 0.029 ± 0.011 (range, 0.016–0.043) for the RapidArc plans. For low‐point dose in the avoidance structures, the CLs were 0.029 ± 0.006 (range, 0.024–0.039) for the IMRT plans and 0.027 ± 0.013 (range, 0.017–0.047) for the RapidArc plans. The average 95% CLs using 3%/3 mm gamma criteria in the high‐dose region were 5.9 ± 2.7 (range, 1.4–8.6) and 3.9 ± 2.9 (range, 1.5–8.8) for IMRT and RapidArc plans, respectively. The average 95% CLs in the low‐dose region were 5.3 ± 2.6 (range, 1.2–7.4) and 3.7 ± 2.8 (range, 1.8–8.3) for IMRT and RapidArc plans, respectively. Based on ion chamber, as well as film measurements, we have established CLs values to ensure the high precision of IMRT and RapidArc delivery for both FF and FFF modalities.

PACS number: 87

## I. INTRODUCTION

TrueBeam linear accelerators (Varian Medical Systems, Palo Alto, CA) have been developed to include both FF (6X, 10X, and 15X) and FFF (6X‐FFF, 10X‐FFF) photon modes for intensity‐modulated radiation therapy (IMRT) and RapidArc (the implementation of VMAT technique on TrueBeam system) treatment delivery. There has been an increasingly interest in the clinical application of these techniques. Several studies have been published to discuss the commissioning procedures and dosimetric characteristics of the TrueBeam system.^1^, ^2^, ^3^ However, there is a clear distinction between commissioning and planning and delivery verification. It is critical to develop QA protocols during commissioning to assure patient safety when using these new technologies.

Volumetric‐modulated arc therapy (VMAT) has been shown as a promising delivery method resulting in plan quality of equal or better than that of IMRT for several sites.^4^, ^5^, ^6^, ^7^ It has gained widespread adoption in the recent years by treating various sites, including prostate, spine, head and neck.^8^, ^9^, ^10^ The dynamic features of VMAT and corresponding optimization constraints are significantly different from the dynamic MLC delivery technique in IMRT.^(11)^ In the progressive resolution optimizer, the optimization algorithm used in RapidArc, the optimization places efficient constraints on variation of continuous variables (MLC aperture and weights) to ensure the continuous delivery, and have four phases with increasing resolution (from 18° to 2°) to converge to the optimal dose distribution.^(12)^ Many factors have an impact on the delivery accuracy in the optimization including sample spacing, gantry rotation speed, maximum leaf motion speed, and dose rate. Commissioning and QA of VMAT have been studied extensively.^13^, ^14^, ^15^, ^16^, ^17^ Kielar et al.^(18)^ evaluated the planning and delivery accuracy of IMRT and RapidArc treatments systematically by optimizing the dosimetric leaf gap (DLG) parameters.

The FFF modality has been introduced to increase dose rate and reduce leaf transmission, head scatter, and leakage radiation with the removal of the flattening filters. The beam‐on time can be reduced significantly for stereotactic body radiation therapy (SBRT) delivery.^(19)^ A sharper penumbra can also be generated from FFF beams.^(20)^ There is a noticeable dose reduction outside of the field in FFF beams compared to FF beams, which can improve the target conformity and have sharper dose drop‐off to limit radiation to distant organs.^(21)^ Even though the dosimetric characteristics of FFF beam have been reported both in experimental and Monte Carlo studies,^22^, ^23^, ^24^, ^25^ the application of FFF beams in the planning and delivery is complicated. The dose rate for FFF modality can be increased up to 2400 MU/min, which can significantly reduce the beam‐on time, thereby limiting the dosimetric impact of intrafractional tumor motion. But the leaf traveling time and speed are limiting factors for the effective and efficient use of FFF beams. Dose rate needs to be modulated (reduced) to deliver the required MU for each segment. The out‐of‐field dose can be reduced with FFF beams with reduced head scattering and leakage. However, in order to achieve uniform dose to the tumor, especially for the large tumors, it needs the distance‐dependent modulation, since the intensity of the beam decreases sharply with the off‐axis distance. This could increase MU and offset the advantage of using FFF beams, as Zhuang et al. reported.^(26)^ However, to date, only limited data have been reported to verify the delivery accuracy of dose calculation of FFF beams for both IMRT and VMAT techniques.^27^, ^28^ FFF beams and arc delivery have been used clinically in helical tomotherapy.^(29)^ However, the slice‐by‐slice delivery of tomotherapy is different from the volumetric delivery of VMAT in many aspects. In tomotherapy, a three‐dimensional (3D) dose distribution is generated by superposing two‐dimensional (2D) dose planes calculated for each 360° rotation. Instead, VMAT calculates volumetric dose distribution in one rotation. The beam in tomotherapy is further collimated into a fan‐beam shape with a width of 2.5 cm by an adjustable jaw, and the intensity is modulated by 64 binary MLC. FFF beams in linac can be generated in any field size and modulated by continuous leaf positioning. Tomotherapy modulates the dose rate by changing MLC opening time, while linac varies the dose rate by adjusting output.

While the new techniques have improved treatment efficiency, they have also increased the complexity of treatment delivery which could potentially affect the accuracy. The Radiological Physics Center's credentialing and retrospective review programs revealed that roughly 30% of institutions had failed to meet the criteria of 7% and/or 4 mm of the 250 irradiations of a head and neck phantom after adoption of IMRT for a decade.^(30)^ Thus, it is important to evaluate the planning and verification accuracy in a more comprehensive manner, and set baseline values during commissioning. There is a lack of commissioning studies that systematically verify that treatments can be planned and delivered accurately using IMRT and VMAT techniques for both FF and FFF photon modes. AAPM TG‐119 has provided confidence limits (CLs) for IMRT commissioning of 6X mode with a set of test cases by combining data from nine participating institutions.^(31)^ The established confidence limit can be used to determine the action level of patient‐specific QA for IMRT treatment. Mancuso et al.^(32)^ compared the action levels of patient specific QA between IMRT and SmartArc (VMAT in Pinnacle) for 6X following the TG‐119 procedures, and showed no statistical difference. A multiinstitutional study was conducted by the European TrueBeam Council to assess the reliability of delivering IMRT and RapidArc patient‐specific plans using FFF mode.^(28)^ It showed that the average gamma result was 98.9% (3 mm, 3%) over 224 patient‐specific plan verifications in four institutions. And the delivery of IMRT and RapidArc plans was equally accurate. With an increasing utilization of FFF beams and RapidArc technique, a systematic dosimetric evaluation similar to TG‐119 should be performed in the commissioning to ensure the quality of treatments.

A TrueBeam system with Millennium 120 MLC was recently commissioned in our institution with all five photon energies. The detailed commissioning procedures have been published.^(2)^ The purpose of this study is to evaluate the overall accuracy of the beam commissioning, and establish CLs for both IMRT and RapidArc of all five photon energies in the TrueBeam system, based on the TG‐119 guidelines.

## II. MATERIALS AND METHODS

### A. Evaluation of MLC performance

The following methodology was used to evaluate the effects of gantry range, gantry speed, leaf speed, and dose rate on MLC positioning and to test the error detection capability of the system. Tests were designed to replicate the work originally proposed by Ling et al.,^(17)^ and all incorporated RapidArc test plans and QA files were provided by Varian Medical Systems. A series of Picket Fence (PF) tests were measured using films for FFF beams and the electronic portal imaging device (EPID) for FF beams. Static PF tests were performed at four cardinal gantry angles to evaluate gantry angle dependence. The full width half maximum (FWHM) values were compared between profiles of leaf match lines. Dynamic PF images acquired using a RapidArc technique were also compared with static PF images and profiles were measured across the 1.0 mm PF gaps. PF tests during RapidArc with an intentional error of 0.5 mm were delivered to evaluate whether submillimeter errors could be detected. A combination of seven dose rates and gantry speed (DR_GS), as shown in Table 1, were delivered to evaluate the control accuracy during RapidArc delivery. Equal dose was given to each of seven 1.8 cm wide strips using a different DR_GS. The deviation for each DR_GS combination was calculated by taking the ratio of the reading to the average value for all the positions.

(1) $$R_{corr}(x)=\frac{R(x)}{R_{open}(x)}\cdot 100$$

where, $R_{corr}(x)$ is the corrected reading at each strip, R(x) is a PF test image reading, $R_{open}(x)$ is an open field $\left(14\;{\text{cm}}^{2}\;\times \;40\;{\text{cm}}^{2}\right)$ image reading. Sixty (60) MU was delivered to the open field, as well as each strip. Similarly, accurate control of the MLC leaf speed was evaluated by delivering four strips of equal dose, but differing combinations of leaf speed and dose rate (LS_DR) using the same methodology. The output was also measured using a PinPoint chamber (PTW, Freiburg, Germany, model 31014) in a Lucy phantom (Standard Imaging, Middleton, WI) at the four cardinal gantry angles.

**Table 1:** The combinations of DR_GS tested for RapidArc delivery

	*FF Beams*		*FFF Beams*	
*Gantry Range (deg)*	*DR* (MU/min)	*GS* (deg/s)	*DR* (MU/min)	*GS* (deg/s)
179.0				
169.0	241.9	4.8	241.9	4.8
86.5	175.9	4.8	175.9	4.8
76.5	241.9	4.8	241.9	4.8
35.5	351.9	4.8	351.9	4.8
25.3	241.9	4.8	241.9	4.8
357.8	527.8	4.8	527.8	4.8
347.8	241.9	4.8	241.9	4.8
327.1	600.0	4.1	703.9	4.8
317.1	241.9	4.8	241.9	4.8
300.6	600.0	3.3	879.7	4.8
290.6	241.9	4.8	241.9	4.8
276.0	600.0	2.9	989.4	4.8
266.0	241.9	4.8	241.9	4.8
252.8	600.0	2.6	1099.6	4.8
242.8	241.9	4.8	241.9	4.8

The EPID sensitivity depends on dose rate and dose. It cannot be used to evaluate the dose pattern due to the saturation effects and the large variation of signal through the radiation field for FFF beams. Therefore, EPID was used to perform the tests for the FF beams and EBT3 films were used for FFF beams. The images taken from EPID were analyzed in the portal dosimetry (Varian Medical Systems). The images irradiated on EBT3 films were analyzed with internal developed software for film dosimetry.

### B. Leaf transmission and dosimetric leaf gap

Since the dosimetric accuracy of IMRT and RapidArc is highly sensitive to the leaf transmission and DLG, a hybrid approach was performed to optimize the settings of leaf transmission and DLG. The baseline values were measured according to the manufacturer's guidelines. Leaf transmission was measured for both leaf banks using a CC13 chamber (Scanditronix Wellhofer, IBA Dosimetry America, Bartlett, TN). The ion chamber was set up at 90 cm SSD and 10 cm depth in water. Five positions with 2 mm separation were measured for each bank to average out the intraleaf transmission. Similarly, DLG was measured under the same setup with the same chamber for a variety of sliding MLC gap widths (1, 2, 4, 6, 10, 14, and 20 mm) spanning 120 mm at a constant speed 0.73cm/s according to the manufacturer's guidelines. However, since only a single DLG parameter is modeled for different field sizes and delivery techniques in Eclipse (Varian Medical Systems), it could induce the modeling inaccuracy. Therefore, the combinations of DLG and transmission were further optimized in Eclipse, based on the agreement between ion chamber and film measurement results and the calculations in Eclipse for all TG‐119 plans.

### C. Couch model

For the Exact IGRT couch associated with TrueBeam, Eclipse can model the thin, medium, and thick portion of the couch. According to the indexing numbers on the couch, the thick portions of the couch is used between F8–F1 for pelvis, the medium couch between F1‐H2 for thorax, and the thin couch between H2‐H4 for H&N. According to previous Monte Carlo and empirical studies, the CT (HU) value of the surface shell was changed to “‐700”, and interior structure to “‐960”.^(33)^ The “thin” and “thick” couch transmissions were measured using the PTW chamber inserted in the center of a Lucy phantom. The phantom was irradiated with a PA field at three different locations, one where the phantom was extended over the couch so that there was no couch in the field, and at both the thin and thick portions of the couch.

### D. TG‐119 IMRT and RapidArc dosimetric verification

A virtual water phantom (Standard Imaging) consisting of multiple solid water slabs was used for planning and measurement purposes. The phantom was water‐equivalent with a density of $1.03\;g/{\text{cm}}^{3}$ and dimensions of $30\;\times \;30\;\times \;15\;{\text{cm}}^{3}$. One slab of the phantom had a drilled cavity for a PinPoint ion chamber (inner diameter 2 mm, volume $0.015\;{\text{cm}}^{3}$).

**Figure 1:**
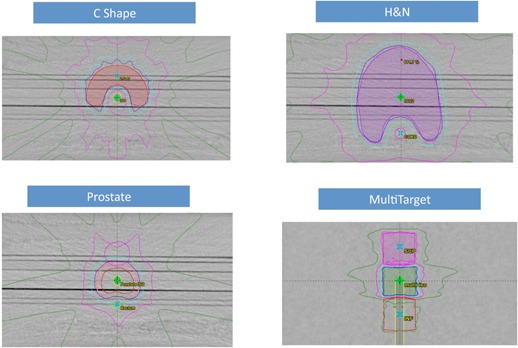
C‐shape target planned with IMRT using 10X‐FFF; H&N PTV target with the cord and parotid glands planned with IMRT using 10X; prostate PTV target with rectum and bladder planned with IMRT using 6X‐FFF; MultiTarget with three cylindrical targets planned with RapidArc using 6X‐FFF. The isodose lines represent 95% (blue), 80% (cyan), 50% (magenta), and 25% (green) prescription dose.

Treatment plans were calculated with the anisotropic analytical algorithm (AAA, V.10028) in the Eclipse treatment planning system. The test plans represented common clinical treatment sites as described in the TG‐119 report.^(31)^ These tests included: AP/PA open fields, bands, multiple target, prostate, head & neck, easy C‐shape, and hard C‐shape. The structures were downloaded as DICOM‐RT data from the AAPM server. For the 6X‐FFF and 10X‐FFF modalities, the dose rates were 1400 and 2400 MU/min, respectively, while for other three energies, the dose rates were 400 MU/min for IMRT plans and the maximum dose rate was set to 600 MU/min for RapidArc plans. Table 2 lists the structures, and IMRT beam arrangement, as well as the measurement location, following the TG‐119 guideline. One arc was used in the RapidArc planning for all cases. All the cases were planned based on TG‐119 criteria and the Exact IGRT couch was included in the dose calculation. Figure 1 shows the isodose distribution of sampled cases in the coronal view (C‐shape, H&N and prostate) and axial view (MultiTarget) at isocenter.

For all cases except the “bands”, one ion chamber measurement (PTW PinPoint Chamber) was made in the target and the other in a low‐dose region. The ion chamber calibration was performed by irradiating the phantom with 100 MU per field, using parallel‐opposed $10\;\times \;10\;{\text{cm}}^{2}$ AP/PA fields arranged isocentrically. The conversion factor from ionization to dose was established using the ratio of the reading and the dose from the treatment planning system. The calibration factor was established by averaging the two conversion factors of the AP/PA fields. The purpose was to reduce the effects of daily output variations and differences of stopping power and mass energy absorption coefficient between solid water and liquid water. To validate the calibration factor, the PinPoint chamber was also cross‐calibrated with an A12 chamber following the recommendation of TG‐51. The deviations between the two calibration methods were around 2% due to the reasons mentioned above. Similarly, two GAFCHROMIC EBT3 films were placed in the coronal planes to measure planar dose distributions in the high‐ and low‐dose regions.

**Table 2:** The test cases and corresponding structures, measurement locations, and IMRT beam arrangements following TG‐119 guideline

*Test Cases*	*Structures*	*Target*	*The Avoidance Structure*	*Beam Arrangement*
AP/PA	$10\;\times \;10\;{\text{cm}}^{2}$ open fields	Midphantom	N/A	AP/PA
Band	A set of five bands	Midphantom	N/A	AP/PA
MultiTarget	3 cylindrical targets	Midphantom	Midphantom	7 fields at 50° intervals
Prostate	PTV, Rectum, Bladder	Midphantom (PTV)	2.5 cm posterior to isocenter (rectum)	7 fields at 50° intervals
Head and Neck	PTV, Parotid glands, spinal cord,	Midphantom (PTV)	4.0 cm Posterior to isocenter (cord)	9 fields at 40° intervals
C‐shape	C‐shape PTV, center core	Midphantom (core)	2.5 cm anterior to isocenter (PTV)	9 fields at 40° intervals

### E. Film dosimetry

GAFCHROMIC film has excellent spatial resolution and is independent of beam angle, energy and dose rate.^(34)^ This makes it suitable for a variety of commissioning and treatment plan QA tasks, especially for FFF beams, as dose rate is modulated for RapidArc delivery. We have developed an efficient film dosimetry protocol that converts optical density values to dose, establishes a calibration curve, registers film and planar dose, and analyzes profile and gamma values. Calibration films were irradiated for each photon energy with a nine $2\;\times \;2\;{\text{cm}}^{2}$ square dose pattern ranging from 0.4 to 3.2 Gy. The sampled optical density values of each color channel were then paired with the calculated dose values to establish the calibration curve through a cubic polynomial least squares fitting. The wait time from irradiation to scanning was approximately 24 hrs for postirradiation coloration. An Epson Expression 10000XL document flatbed scanner (Seiko Epson Corp, Nagano, Japan) with Epson Scansoftware was used to scan the films. The scanner was warmed up by 16 consecutive blank scans before each scanning session.^35^, ^36^ Each film was scanned in the center of the scanner bed to allow for better scanner response uniformity. The films were scanned in transmission mode for better scanning stability with settings of 75 dot‐per‐inch and 48 bit RGB mode (16 bits per color channel). Images were exported in tagged image file format (TIFF) for analysis and image processing filters were disabled.

### F. Statistics

Dose difference ratios and CLs were calculated following TG‐119's definition. The difference between measurement and calculation was defined as (measured dose ‐ plan dose)/prescription dose. The concept of confidence limit has been introduced to evaluate the influence of systematic and random deviations by Venselaar et al.^(37)^ The confidence limit is defined as |mean| + 1.96 σ for point dose measurements. Mean (systematic difference) and standard deviation (random difference) were calculated for both high‐ and low‐dose points with each technique (IMRT or RapidArc) and energy. The factor 1.96 is derived from the normal distribution so that 95% of the data should fall within the confidence limit. Using the same approach, the confidence limit was also calculated for passing gamma criteria of 3%/3 mm for both high‐ and low‐dose planes. The formulation was defined as 100‐mean + 1.96 σ.

## III. RESULTS

### A. MLC performance

**Figure 2:**
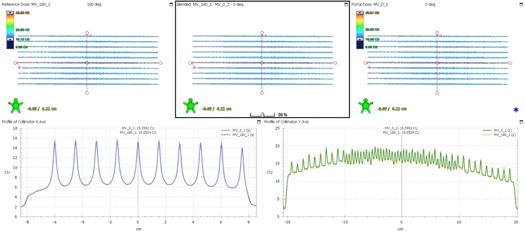
The PF test images comparison at two different gantry angles for 6X. The PF image taken at gantry 180° is shown in the upper left. The image taken at gantry 0° is shown in the upper right. The blended one is shown in the upper middle. The profiles across the fence from the PF images at gantry 0° (solid blue line) and 180° (dashed red line) are shown in the lower left. The profiles along the fence from the PF images at gantry 0° (solid green line) and 180° (dashed orange line) are shown in the lower right.

**Figure 3:**
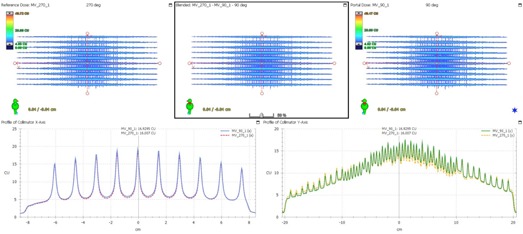
The PF test images comparison at two different gantry angles for 6X‐FFF. The PF image taken at gantry 270° is shown in the upper left. The image taken at gantry 90° is shown in the upper right. The blended one is shown in the upper middle. The profiles across the fence from the PF images at gantry 90° (solid blue line) and 270° (dashed red line) are shown in the lower left. The profiles along the fence from the PF images at gantry 90° (solid green line) and 270° (dashed orange line) are shown in the lower right.

**Figure 4:**
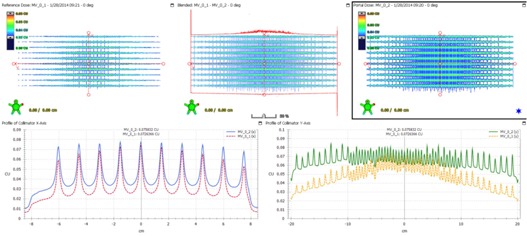
The PF test images comparison between 6X and 6X‐FFF beams at gantry angle 0°. The PF image for 6X‐FFF is shown in the upper left. The image for 6X is shown in the upper right. The blended one is shown in the upper middle. The profiles across the fence from the PF images for 6X (solid blue line) and 6X‐FFF (dashed red line) are shown in the lower left. The profiles along the fence from the PF images for 6X (solid green line) and 6X‐FFF (dashed orange line) are shown in the lower right.

The effect of gantry angle/rotation on leaf accuracy was evaluated using the EPID for FF mode and EBT3 films for FFF mode. The Picket Fence image at each gantry angle was compared (overlaid) with each other. The Picket Fence image at gantry 0° was also compared between 6X and 6X‐FFF mode. Cross‐plane and in‐plane profiles were drawn and the locations of the profile peaks were within 1 mm tolerance. Figure 2 shows the comparison of the PF images for 6X at gantry 0° and 180°. Figure 3 shows a similar comparison for 6X‐FFF at gantry 90° and 270°. Figure 4 shows the comparison of the PF images between 6X and 6X‐FFF images at gantry 0°. The dynamic PF image was also overlaid with the static PF image to evaluate the leaf motion accuracy of RapidArc delivery. The locations of the profile peaks were also within 1 mm tolerance.

**Figure 5:**
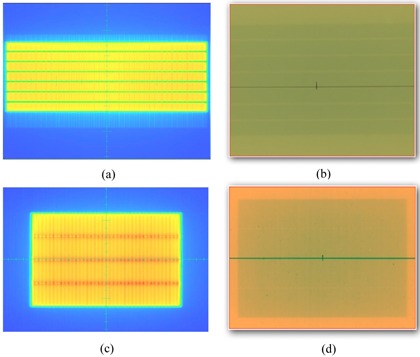
The test images to evaluate the accurate control of dose rate and gantry speed (DR_GS) during RapidArc delivery by delivering equal doses to seven 1.8 cm wide strips, with varying combinations of dose rate, gantry range and gantry captured (a) with EPID for 6X, (b) with EBT3 film for 6X‐FFF. The test images to evaluate the accurate control of the MLC leaf speed during RapidArc delivery by delivering four strips of equal dose but differing combinations of leaf speed and dose rate (LS_DR) captured (c) with EPID for 6X, (d) with EBT3 film for 6X‐FFF.

The intentional induced 0.5 mm positional errors were easily discerned on both EPID and film images. Table 3 lists the dose deviation calculation with seven combinations of DR_GS for 6X and 6X‐FFF and Table 4 for the leaf speed deviation calculation. Each position in the tables corresponds to a region of interest defined in one of the strips, delivered with a different DR_GS or LS_DR. Figure 5 shows the images for DR_GS tests with EPID for 6X (a), and with EBT3 film for 6X‐FFF (b), and for LS_DR tests with EPID for 6X (c), and with EBT3 film for 6X‐FFF (d). The mean values and ranges of the absolute dose deviation for variable DR_GS and LS_DR tests during RapidArc delivery are listed in Table 5. The output was within 0.8% at the four cardinal gantry angles.

**Table 3:** DR_GS tests for RapidArc delivery of 6X and 6X‐FFF modes. CU stands for calibrated unit. OD stands for optical density. The corrected reading at all positions was calculated using the formula $R_{corr}\left(x\right)\;=\;\frac{R\left(x\right)}{R_{open}\left(x\right)}\;ċ\;100$. The deviation for each DR_GS combination was calculated by taking the ratio of the reading to the average value for all the positions

*6X*	DR_GS						
Position (cm)	‐5	‐3	‐1	1	3	5	7
Mean (CU)	74.06	74.18	73.95	73.96	73.81	73.87	71.73
SD	0.42	0.37	0.42	0.37	0.39	0.49	0.57
	*Open field*						
Mean (CU)	68.38	69.32	69.28	69.42	69.38	69.38	67.32
SD	0.16	0.08	0.07	0.09	0.08	0.10	0.25
R_corr	108.31	107.01	106.74	106.54	106.39	106.47	106.55
R_corr mean				106.86			
Diff (%)	1.4	0.1	‐0.1	‐0.3	‐0.4	‐0.4	‐0.3
Pass?	YES	YES	YES	YES	YES	YES	YES
*6X‐FFF*				DR_GS			
Position (cm)	‐5	‐3	‐1	1	3	5	7
Mean (OD)	38354.50	37602.75	37124.70	37209.20	37278.40	38009.60	38792.80
SD	71.70	86.40	63.90	58.00	90.70	111.70	91.60
	*Open field*						
Mean (OD)	39924.80	39358.10	39047.50	38985.40	39145.20	39614.00	40215.60
SD	152.60	113.20	103.10	115.00	97.00	219.90	118.00
R_corr	96.07	95.54	95.08	95.44	95.23	95.95	96.46
R_corr mean				95.68			
Diff (%)	0.4	‐0.2	‐0.6	‐0.3	‐0.5	0.3	0.8
Pass?	YES	YES	YES	YES	YES	YES	YES

**Table 4:** LS_DR tests for RapidArc delivery of 6X and 6X‐FFF modes. The unit of mean values is CU for 6X and OD for 6X‐FFF. The corrected reading at all positions was calculated using the formula $R_{corr}\left(x\right)\;=\;\frac{R_{LS}\left(x\right)}{R_{open}\left(x\right)}\;ċ\;100$. The deviation for each point was calculated by taking the ratio of the reading to the average value for all the positions

	*6X*				*6X‐FFF*			
Position (cm)	‐4.5	‐1.5	1.5	4.5	‐4.5	‐1.5	1.5	4.5
Mean	37.37	37.63	37.09	36.15	41931.7	41521.8	41499.2	41884.7
SD	0.37	0.96	0.34	0.4	122.7	110.4	88	122.3
*Open*								
Mean	67.12	67.34	66.78	65.32	40158.9	39626.7	39533.6	40153.9
SD	0.11	0.2	0.06	0.16	196.1	119.6	120	158.6
R_corr	55.68	55.88	55.54	55.34	104.41	104.78	104.97	104.31
R_corr mean		55.61				104.62		
Diff (%)	0.1	0.5	‐0.1	‐0.5	‐0.2	0.2	0.3	‐0.3
Pass?	YES	YES	YES	YES	YES	YES	YES	YES

**Table 5:** The mean values and ranges of the absolute dose deviation for variable DR_GS and LS_DR tests during RapidArc delivery

	*6X*	*10X*	*15X*	*6X‐FFF*	*10X‐FFF*
Mean (DR_GS)	0.43%	0.41%	0.30%	0.43%	0.51%
Range (DR_GS)	‐0.44%–1.36%	‐0.42%–1.13%	‐0.31%–1.02%	‐0.63%–1.82%	‐0.79%–1.02%
Mean (LS_DR)	0.30%	0.38%	0.36%	0.25%	0.24%
Range (LS_DR)	‐0.48%‐0.49%	‐0.71%–0.53%	‐0.67%–0.48%	‐0.30%–0.34%	‐0.26%–0.26%

### B. Leaf transmission and DLG

Table 6 lists the DLG and MLC transmission for all photon energies. The measured values were acquired following the procedures recommended by the vendor. The values on this Linac were fine‐tuned, based on the ion chamber measurements of the TG‐119 plans using RapidArc techniques. The adjustment of DLG was significant, ranging from 0.25 (6X) to 0.70 (15X) mm. The adjustment of transmission was within 0.23%.

**Table 6:** The measured and optimized DLG and MLC transmission values for all photon energies

*Energy*	*6X*	*10X*	*15X*	*6X‐FFF*	*10X‐FFF*
DLG (mm) ‐ Measured	0.95	1.10	1.10	0.80	1.01
DLG (mm) ‐ Optimized	1.20	1.60	1.80	1.20	1.60
Transmission ‐ Measured	1.52%	1.56%	1.69%	1.29%	1.73%
Transmission ‐ Optimized	1.52%	1.73%	1.69%	1.52%	1.56%

### C. Couch transmission

Table 7 summarizes the calculated and measured transmission value of both thin and thick components of the couch for each energy. The calculated transmission value for each energy was in the range of 0.9733 to 0.9846 for the “thick” couch, and 0.9794 to 0.9877 for the “thin” couch. The average differences between the calculated and measured values were −0.50% and −0.78% for the “thin” and “thick” couches, respectively.

**Table 7:** Transmission factors of Exact IGRT couch

	*“Thick” Couch Transmission*			*“Thin” Couch Transmission*	
	*Calculated*	*Measured*	*%Diff*	*Calculated*	*Measured*	*%Diff*
6X	0.9767	0.9682	‐0.9	0.9825	0.9744	‐0.8
10X	0.9733	0.9630	‐1.1	0.9794	0.9723	‐0.7
15X	0.9829	0.9763	‐0.7	0.9861	0.9829	‐0.3
6X‐FFF	0.9791	0.9727	‐0.7	0.9835	0.9799	‐0.4
10X‐FFF	0.9846	0.9784	‐0.6	0.9877	0.9851	‐0.3

## D. TG‐119 measurements

### D.1 Ion chamber measurement results

8, 9 show the ion chamber measurement results in the high‐dose and low‐dose regions, respectively, for both IMRT and RapidArc plans. Dose difference ratio and CLs were calculated following TG‐119's definition. For the high‐dose low‐gradient target, the dose difference ratio was ‐0.002 ± 0.015 (range, −0.031–0.021) in the IMRT plans and 0.008 ± 0.012 (range, −0.009–0.033) in the RapidArc plans, corresponding to the average 95% CLs of 0.030 ± 0.007 (range for each energy, 0.022–0.038) and 0.029 ± 0.011 (range for each energy, 0.016–0.043), respectively. The CLs were within 0.045, which was the average CLs over all tests and institutions for the high‐dose region from TG‐119. For the low‐dose point in the avoidance structures, the dose difference ratio was 0.001 ± 0.014 (range, −0.025–0.025) in the IMRT plans and 0.008 ± 0.011 (range, −0.016–0.047) in the RapidArc plans, corresponding to CLs of 0.029 ± 0.006 (range for each energy, 0.024–0.039) and 0.027 ± 0.013 (range for each energy, 0.017–0.047) respectively. The CLs were within 0.047, the average CLs over all tests and institutions in low‐dose region from TG‐119.

**Table 8:** High‐dose point in the PTV measured with ion chamber for both IMRT and RapidArc plans. The difference between measurement and calculation was defined as [(measured dose‐plan dose)/prescription dose]

	*IMRT*					*RapidArc*				
	*6X*	*10X*	*15X*	*6X‐FFF*	*10X‐FFF*	*6X*	*10X*	*15X*	*6X‐FFF*	*10X‐FFF*
Prostate	1.5%	0.0%	0.1%	1.8%	‐1.6%	0.8%	0.0%	‐0.2%	‐0.3%	‐0.9%
H&N	0.5%	‐1.9%	‐2.2%	2.1%	‐3.1%	0.3%	‐0.3%	‐0.1%	0.2%	0.1%
C‐shape hard	0.1%	‐0.9%	‐2.1%	0.4%	‐0.7%	3.3%	1.5%	1.0%	2.3%	‐1.0%
C‐shape easy	1.0%	‐1.3%	‐2.1%	1.4%	0.2%	2.9%	1.5%	0.0%	1.2%	3.1%
MultiTarget	1.6%	‐0.0%	‐0.0%	1.9%	‐1.8%	1.1%	0.4%	1.1%	1.0%	0.4%
Overall Mean	0.9%	‐0.8%	‐1.3%	1.5%	‐1.4%	1.7%	0.6%	0.4%	0.9%	0.3%
Overall SD	0.007	0.008	0.012	0.007	0.012	0.014	0.008	0.006	0.01	0.016
Confidence Limit	0.022	0.024	0.036	0.029	0.038	0.043	0.023	0.016	0.028	0.035

**Table 9:** Low‐dose point in the avoidance structure measured with ion chamber for both IMRT and RapidArc plans. The difference between measurement and calculation was defined as [(measured dose‐plan dose)/prescription dose]

	*IMRT*					*RapidArc*				
	*6X*	*10X*	*15X*	*6X‐FFF*	*10X‐FFF*	*6X*	*10X*	*15X*	*6X‐FFF*	*10X‐FFF*
Prostate	2.5%	‐0.1%	0.0%	2.1%	‐1.6%	4.7%	2.0%	1.3%	2.1%	0.6%
H&N	0.1%	‐1.1%	‐1.7%	1.4%	‐1.3%	2.0%	1.2%	1.0%	1.7%	1.1%
C‐shape hard	1.9%	1.8%	1.0%	0.0%	1.3%	0.7%	0.4%	‐0.4%	‐1.6%	‐0.6%
C‐shape easy	0.7%	‐0.4%	‐0.6%	0.1%	0.7%	0.4%	0.4%	‐0.1%	0.5%	1.1%
MultiTarget (Sup)	1.2%	‐0.9%	‐1.3%	1.4%	‐2.5%	1.1%	0.8%	0.7%	0.3%	‐0.3%
MultiTarget (Inf)	1.4%	‐1.0%	‐1.5%	1.3%	‐1.8%	0.8%	0.3%	‐0.1%	0.2%	0.1%
Overall Mean	1.3%	‐0.3%	‐0.7%	1.0%	‐0.9%	0.016	0.009	0.004	0.005	0.003
Overall SD	0.008	0.011	0.01	0.008	0.015	0.016	0.007	0.007	0.013	0.007
Confidence Limit	0.029	0.024	0.027	0.027	0.039	0.047	0.022	0.018	0.031	0.017

### D.2 Film measurement results

**Figure 6:**
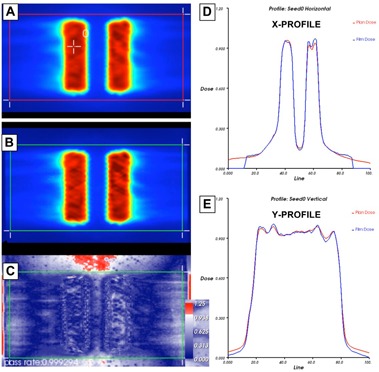
Planned axial dose distribution (a) at central core level (i.e., low‐dose region) of 6X hard C‐shape plan using RA technique; (b) exposed EBT3 film at the same level; (c) film gamma analysis results with 3%/3 mm criteria (99.9% of pixels passed within region of interest); the corresponding X (d) and Y (e) profiles for planned and delivered film doses. The red line indicates planned dose, whereas the blue line indicates the measured dose.

Figure 6 shows an example of film analysis in the low‐dose region of the 6X, Hard C‐Shape RapidArc plan. 10, 11 show the percentage of points passing the recommended 3%/3 mm gamma criteria in the high‐dose and low‐dose regions, respectively, for both IMRT and RapidArc plans. The percentage of points passing the gamma criteria, averaged over all tests was 98.0 ± 2.2 (IMRT) and 98.7 ± 1.8 (RapidArc) for the high‐dose plans, 98.5 ± 1.8 (IMRT) and 99.0 ± 1.6 (RapidArc) for the low‐dose planes. The CLs using 3%/3 mm gamma criteria were 5.9 ± 2.7 (range, 1.4–8.6) for IMRT and 3.9 ± 2.9 (range, 1.5–8.8) for RapidArc in the high‐dose planes, 5.3 ± 2.6 (range, 1.2–7.4) for IMRT and 3.7 ± 2.8 (range, 1.8–8.3) for RapidArc in the low‐dose planes.

**Table 10:** Gamma passing rate (3%/3 mm) in the high‐dose PTV plane with associated confidence limits for both IMRT and RapidArc plans

	*IMRT*					*RapidArc*				
	*6X*	*10X*	*15X*	*6X‐FFF*	*10X‐FFF*	*6X*	*10X*	*15X*	*6X‐FFF*	*10X‐FFF*
Prostate	96.7	99.5	99.2	99	97.8	100	96.9	99.9	100	98
H&N	97.8	99.3	99.1	93.6	99.4	98.6	98.3	98.5	99.9	99.5
C‐shape hard	99.9	99.5	88.5	96.8	93.9	99.9	99.9	99.4	97.5	92.6
C‐shape easy	99.4	89.2	98.9	98.1	98.5	99.9	98	99.9	99.9	95.1
MultiTarget	92.9	99.3	99	97.4	99.6	100	99.9	98.1	99.5	97.3
Overall Mean	96.8	98.4	99.1	97.2	98.2	99.7	98.6	99.2	99.4	96.5
Overall SD	2.8	2.5	0.3	2.0	2.2	0.6	1.3	0.8	1.1	2.7
Confidence Limit	8.6	6.6	1.4	6.6	6.2	1.5	3.9	2.4	2.7	8.8

**Table 11:** Gamma passing rate (3%/3 mm) in the low‐dose avoidance structure plane with associated confidence limits for both IMRT and RapidArc plans

	*IMRT*					*RapidArc*				
	*6X*	*10X*	*15X*	*6X‐FFF*	*10X‐FFF*	*6X*	*10X*	*15X*	*6X‐FFF*	*10X‐FFF*
Prostate	99.1	99.6	98.1	98.6	99.1	99.9	98.8	100	93.2	99.7
H&N	99.2	99.9	100	99.8	99.8	99.8	99.4	99.8	99.9	99.9
C‐shape hard	99.7	94.2	96.9	99.5	95.4	100	99.9	100	99.9	98.5
C‐shape easy	99.5	99.1	97.9	93.7	89.3	96.6	98.1	100	100	99.9
Overall Mean	99.4	98.2	98.2	97.9	98.2	98.7	98.7	99.6	98.2	99.5
Overall SD	0.3	2.7	1.3	2.1	2.8	1.5	0.6	0.7	3.4	0.7
Confidence Limit	1.2	7.1	4.3	6.3	7.4	4.3	2.5	1.8	8.3	1.8

## IV. DISCUSSION

We present a direct comparison of IMRT and RapidArc plans for all five photon energies using TG‐119 test cases during commissioning. It provided the direct assessment of the quality of commissioning and allowed us to optimize the MLC settings. No statistically significant (p > 0.05) differences were found between FF and FFF beams. FFF beams achieved the same level of CLs as FF beams. There were statistically significant differences between IMRT and RapidArc delivery of 10X(p = 0.024) and 15X(p = 0.028) in the ion chamber measurement. The CLs of RapidArc plans were slightly better than those of IMRT plans, due to the optimization of DLG and transmission based on RapidArc measurement results. The CLs of each technique and energy were well below the baseline values published in TG‐119. This type of examination provides confidence in the accuracy of this new technology before it is implemented in the clinic.

The evaluation of MLC performance was performed based on the standard recommendations, including Picket Fence tests, delivered in both stationary and rotational gantry modes. The radiation pattern relative to that of the corresponding open fields was analyzed for different combinations of DR_GS and MLC speeds. We have previously observed systematic differences in the DLG by comparing four TrueBeam systems across three institutions, even though the standard DICOM files and measurement setup were used.^(2)^ However, even a small difference in the DLG can result in large dosimetric errors. Rangel and Dunscombe^(38)^ evaluated the dosimetric consequences of suboptimal MLC performances. They showed that a 0.3 mm systematic error leads to a 2% change in the equivalent uniform dose. The deviation could stem from various sources, including measurement uncertainty and slight differences in MLC leaf material/design. Therefore, it is important to verify the DLG values by comparing the measured dose and calculated doses. The RapidArc delivery was very sensitive to the DLG and leaf transmission setting. If the unoptimized, measured values of DLG and transmission were used directly in the dose calculation, the discrepancy between calculations and ion chamber measurements in the high‐dose region was within 3% for IMRT, but up to 7% for RapidArc. Kielar et al.^(18)^ also reported that the measured DLG value might not be suitable for Rapidarc and could lead to 5% discrepancies. The combinations of DLG and transmission were optimized based on the ion chamber measurement results of RapidArc delivery, which was similar to the methodology of Chauvet et al.^(39)^ that used sliding slit tests. The measurements systematically underestimated the optimized DLG value. The largest adjustment was 0.7 mm for 15X. After the optimization, the dosimetric discrepancies were significantly reduced in the RapidArc delivery with a total of three measurement points greater than 3% in both high‐and low‐dose regions. The adjustment did not have much impact on the IMRT delivery accuracy. Szpala et al.^(40)^ studied the sensitivity of dose calculation on the DLG values using a sliding window technique. They noticed that a single DLG value (2.0 mm) can be applied as the optimal value for all different widths of the MLC slits, since the dose was integrated as the leafs travel across the chamber as a slit. The DLG values for smaller and larger regions were averaged out.

Several sources of uncertainties associated with the film dosimetry, such as scanner, background, and film uniformity, can impact dosimetric accuracy. We developed in‐house software to integrate GAFCHROMIC film dosimetry protocol using EBT3 films which streamlines a dose pattern delivery for calibration, calibration curve fitting, film scanning in the fixed scanner position, dose mapping from multiple color channels, and profile/gamma analysis. During the IMRT and RapidArc commissioning, the measurements were grouped based on energy. For each energy, the calibration film was irradiated first and the corresponding plans were delivered under sequence for both IMRT and RapidArc. Since all the films were scanned 24 hrs after delivery, the impact of time dependence on the dosimeric results was minimal. The protocol ensures the consistency of film dosimetric results with a clear understanding of the uncertainty (~ 2%) of our film dosimetry protocol based on a systematic study in the clinical practice. The detail will be addressed in a separate paper.

## V. CONCLUSIONS

We evaluated the planning and delivery accuracy using FF and FFF beams, as well as IMRT and RapidArc techniques, using ion chamber and EBT 3 films based on TG‐119 protocol. The measurement results and established CLs values can be used as a baseline to assess the accuracy of patient specific QA to ensure high‐precision delivery of IMRT and RapidArc delivery for both FF and FFF modalities.

## Supporting information

Supplementary Material

Supplementary Material

Supplementary Material
