# Supplementary material for: IMRT and RapidArc commissioning of a TrueBeam linear accelerator using TG‐119 protocol cases
Source: J Appl Clin Med Phys. 2014 Sep 8;15(5):74–88. doi: 10.1120/jacmp.v15i5.4843 (PMC5711094; doi:10.1120/jacmp.v15i5.4843)
Supplement: Supplementary file 3 — Supplementary Material [file ACM2-15-074-s003.doc]

*Associate Editor Comments:*

**The wordings from line 148-157 have close semblance from the paper of Ling et al. The problem which we are having that the way these sentences are written, they give an impression that they are being proposed by you for the first time rather than Ling et al. Appropriate wording changes are needed to avoid any future complaints.**

We are very sorry for not making it clear. We followed the methodology from Ling et al for RapidArc commissioning. We have added the following text at line 142.

The following methodology was used to evaluate the effects of gantry range, gantry speed, leaf speed and dose rate on MLC positioning and to test the error detection capability of the system. Tests were designed to replicate the work originally proposed by Ling et.al ^17^ and all incorporated RapidArc test plans and QA files were provided by Varian Medical Systems.

**In line 330, they have mentioned that IMRT & RapidArc pass rates of 3 & 7% respectively due to DLGs. This seems to be odd and should be investigated. Though measurements with 3 devices in the same/similar setup yielding similar results doe not imply that DLG is correct as it may affect all the measurements in a similar manner. Earlier we had suggested a method of using Sun-Nuclear IMF type of device to rule out any gantry rotation issues. It appears that the authors do not have access to such device.**

**Another method which can be done is to create a verification plan of a VMAT case by forcing gantry to remain stationary [Eclipse supports it]. Thus the entire arc is delivered in a stationery manner similar to IMRT delivery.**

**Now make 2 plans with DLG values from static measurements as well as authors determined values and compare the results.**

We agree with the concern that the DLG value could lead to large dose discrepancy in the RapidArc plans should be carefully investigated. We could like to take any recommended measurements to confirm it. We are sorry that we currently do not have Sun-Nuclear IMF device available to repeat the measurement to rule out the gantry rotation related issues.

It is a great idea to create a verification plan of a case by forcing gantry to remain stationary. We tried to work on the case with the largest discrepancy ~6.8% between measurement and calculation before the DLG value adjustment. The fluence could be created in Eclipse at a fixed gantry angle. However, they were not deliverable. The errors were associated with gantry speed and gantry acceleration since the control points could not be broken up among gantry angle, MLC and dose rate. We also contacted Varian and they gave me same answers.
