# Supplementary material for: IMRT and RapidArc commissioning of a TrueBeam linear accelerator using TG‐119 protocol cases
Source: J Appl Clin Med Phys. 2014 Sep 8;15(5):74–88. doi: 10.1120/jacmp.v15i5.4843 (PMC5711094; doi:10.1120/jacmp.v15i5.4843)
Supplement: Supplementary file 1 — Supplementary Material [file ACM2-15-074-s001.doc]

**Point-by-point responses**

We would like to extend our sincere gratitude to the associate editor and the reviewers for their comprehensive critiques, which have helped improve the quality of this manuscript. Below we provide a point-by-point response to the reviewers’ questions and comments.

*Associate Editor Comments:*

General comments:

1. **The title of this paper is very misleading. EBT 3 is hardly discussed in a scientific manner here. I suggest either authors change the title or introduce some more details of it. This work is more of performance evaluation of Truebeam using TG-119 and paper's title should indicate that.**

Sorry for the misleading. We have changed the title to “IMRT and RapidArc Commissioning of TrueBeam Linear Accelerator”. Initially we included the film dosimetry component in the manuscript and realized that it could lead to a split objective for Linac commissioning and the utilization of the EBT films. Therefore we have removed the details of our film dosimetry program. Our integrated film dosimetry protocol was established to streamline a nine dose pattern delivery for dose calibration, calibration curve fitting, dose mapping from multiple color channels, and profile/gamma analysis. To quantify EBT3 film dosimetric uncertainties, films from eight packets were irradiated with open fields and standard deviations of dose maps were calculated at each color channel for evaluation. To determine the temporal dependence of EBT3 films, the average correction factors of different dose levels as a function of time were evaluated up to four days after irradiation. However, we have included some technical details about film calibration, scanning etc in the method.

2. **I am having problem with the following statement of authors "While one could borrow the same CLs for IMRT commissioning of other FF photon energies, it is advantageous to develop new CLs for FFF photon modes and RapidArc during commissioning since the energies and delivery techniques are significant different from the previous ones." The day we will come to know that we need to relax CL for FFF or RapidArc plans because of inability of linac to deliver them, both will not be favored for treatment delivery.**

**In USA, class action lawsuits will follow immediately. A dose is a dose whether it is delivered in FF or FFF mode or by IMRT or VMAT method. Your results show that there is virtually no difference in both when gamma evaluation criterion was used. The reasons of the difference in the CL values has not been dealt in the manuscript.**

We thank the associate editor for the very thoughtful comment. We could not agree more with the reviewer’s suggestion that relaxing FFF or RapidArc CLs would be unwise. However, before adopting, for FFF or RapidArc, the IMRT CLs, the analysis of the commissioning results had to be completed to be confident in the accuracy of the delivery techniques.

We have removed the statement you quoted. Also the text of the introduction has been modified significantly based on the reviewer’s suggestions. We specifically emphasized that “with an increasing utilization of FFF beams and RapidArc technique, a systematic dosimetric evaluation similar to TG119 should be performed in the commissioning to ensure the quality of treatments.”

The CL values were dominated by the standard deviation term with its multiplier of 1.96. Therefore, even though there was not much difference in gamma results, we could still observe the difference in the CL values.

3. **If the results due MLC speed are fine with 2400 MU/min with FFF, why FF fields IMRT were tested for only 400 MU/min? Generally if the MLC are able to keep up with 2400 MU/min, due you think they are likely to fail in 400 MU/min? If yes, why?**

**The maximum dose rate for FF mode is 600 MU/min.**

Thanks for allowing us to clarify. Our commissioning is based on the dose rate used in the clinic. In our institution, the dose rates of FF fields are 400MU/min in the IMRT plans and 600MU/min (maximum) in the RapidArc plans. FFF beams are used for SBRT planning and the maximum dose rates (1400MU/min for 6XFFF and 2400MU/min for 10XFFF) are used to calculate TG119 cases.

400MU/min dose rate has been used in our institution when Eclipse was commissioned about a decade ago. In order to avoid confusion and make the clinical flow streamlined on all our linacs, it is the only dose rate we used for dose calculation using FF beams. Therefore, all our TG 119 cases were calculated with 400MU/min.

With the same MU settings, the higher the dose rate is, the faster the MLC needs to accelerate and travel. Therefore, the MLC is less likely to fail in 400 MU/min if it is able to keep up with 2400 MU/min. But the leaf traveling time and speed also put constraints for the effective and efficient use of high dose rate. Dose rate sometimes needs to be reduced to deliver the required MU for each segment. So eventually the tests should be evaluated based on a combination of dose rate and leaf speed effect.

4. **The commonly accepted dose difference formula is [(measured dose – plan dose)/Plan dose] and not [(measured dose - plan dose)/Prescription dose].**

Thanks for pointing it out. Yes, we agree that the denominator of the f	ormula should be the plan dose in general. However, it was expressed as a ratio of the prescription dose in TG119. According to TG119, the prescription dose was more clinically relevant, especially for low dose regions, for which reporting the difference from the prescription dose can overstate the clinical importance of the deviation.

5. **Why not compare FF & FFF PF test also at the same gantry angles? If the results between both at any given gantry angle are same, it could be expected that the final dose delivery errors should also be very similar.**

Thanks for the suggestion. We have followed your recommendation to compare FF and FFF PF tests. The result was shown in Fig.4. The locations of the profile peaks were also within 1mm tolerance. Due to the difference of the energy spectrum between FF and FFF, the scatter contribution from FFF beam was less and therefore peak values were also low.

6. **The adjustment of DLG was significant, ranging from 0.25 (6X) to 0.70 (15X) mm. Have authors compared these values with those published in literature? Our own measured values for both different than yours and for 15X the difference was very high. Moreover, this method (optimizing based on RapidArc measurements] is not standard and not published before and I am not sure whether people use it? Our DLG measurements for Truebeam have been done by a regular method and our pre-treatment QA results are fine.**

Yes, we agree that optimizing DLG is not a standard way for commissioning. There has been debate regarding how to model the

transmission and the DLG parameter in dose calculations. The modeling inaccuracy has been noted to potentially be caused by the overlap between opposing leaves. In the Eclipse TPS, only a single DLG parameter is modelled for different field sizes and delivery techniques and it is likely to induce the modeling inaccuracy. Actually several papers have been published to discuss the optimization of MLC parameters (Kielar et al, Med. Phys. 39 (10), October 2012 and Chauvet et al., Phys. Med. Biol. 50 (2005)).

We also performed across comparison of the DLG values with other institutions. In a multi-institutional study (including our institution) of commissioning five Truebeams published by Glide-Hurst et al, The average MLC DLGs for 6, 10, and 15 MV photons were 1.33 ± 0.23, 1.57 ± 0.24, and 1.61 ± 0.26 mm, respectively. 6XFFF and 10XFFF modes had average DLGs of 1.16 ± 0.22 and 1.44 ± 0.30 mm, respectively. The number we optimized for 15X was in the higher end but still within a reasonable range.

7. **Couch transmission values given in line 225 with OUT mention of energy are very misleading.**

Sorry for the misleading statement. We have rephrased the statement as “the calculated transmission value for each energy was in the range of 0.9733 and to 0.9846 for the “thick” couch, 0.9794 and to 0.9877 for the “thin” couch”.

8. **In line 245, why such a huge diff in CL but not in gamma?**

The confidence limit is defined as (│mean│+ 1.96σ) for point dose measurements. The factor 1.96 is derived defined based on from the normal distribution so that 95% of the data should fall within the confidence limit. 95% confidence interval reflects a large difference in CL even though the overall mean and standard deviation are not significant.

9. **Line 295, please write the results from ref 16?**

We have provided the results from the reference. “It showed that the average gamma result was 98.9% (3mm, 3%) over 224 patient specific plan verifications in four institutions. And the delivery of IMRT and RapidArc plans was equally accurate.”

10. **Line 310, More confidence in these results could have been recd if both EPID & EBT3 would have been used for FF fields. This would have provided confidence in the film dosimetry by an independent detector.**

This is an excellent point. Actually in order to develop our film dosimetry program, we did cross comparison between EPID and EBT3 on the Novalix TX platform using TG 119 cases. The following table listed the gamma results of four RapidArc plans measured with both EBT3 and EPID for two energies. They had shown very good agreement and provided confidence in our film dosimetry program. Therefore we did not use the portal dosimetry for TG119 commissioning on the TrueBeam. We would like to submit a separate paper to discuss our film dosimetry program in detail.

|  | 6X | | 18X | |
| --- | --- | --- | --- | --- |
|  | EBT3 | EPID | EBT3 | EPID |
| Prostate | 99.9 | 100.0 | 99.6 | 100.0 |
| MultiTarget | 99.8 | 100.0 | 99.6 | 99.9 |
| CShape hard | 99.9 | 100.0 | 100.0 | 100.0 |
| HN | 99.9 | 99.4 | 99.9 | 99.9 |

The figures below are the portal dosimetry and film dosimetry results for a C Shape Hard case delivered by RapidArc technique using 6X.

11. **If might be good idea to use a planar array like [Mapcheck etc.] for comparison purposes as its diode detectors are very small in size. Even pinpoint chamber could be considered big if used in high gradient area.**

We agree with the associate editor that a planar array is also a good option to measure dose fluence map. The active detector area of SunPoint Diode detector used in the Mapcheck is only 0.64 mm, which is very good to measure high dose gradient area. However, we only have Matrixx in our institution instead. The ion chamber diameter is 4.5 mm, which is larger than PinPoint chamber. Gafchromic film has excellent spatial resolution and is independent of beam angle, energy and dose rate. The uncertainty of the films is under 2% based on our protocol and we also validated the accuracy of the film dosimetry by comparing against the portal dosimetry results as discussed above. Therefore we believe we have a robust verification mechanism to evaluate the overall accuracy of IMRT and RapidArc delivery.

*Reviewer C*

General comments:

**This paper is to evaluate the overall accuracy of IMRT and RapidArc delivery using both FF and FFF at a Varian TrueBeam commissioning and therefore present the patient-specific QA baselines following the guidelines of TG-119. The paper is well written and could be of interest to the medical physics community. However, there are many deficits of the paper, as highlighted in the specific comments below. In my opinion, this paper needs a major revision before it can be considered for publication in JACMP.**

Thank you for your insightful comments on this work.

Specific comments:

**Line 61 "intensity modulated" changes to "intensity-modulated".**

Corrected.

**Line 63: "...equal or better that of IMRT for several sites1" - However, the reference#1 was just a comparison study for 12 H&N patients, not "several sites" as the authors stated. I suggest that the authors either add more references for other sites or just mentioned "H&N" in the sentence.**

Thanks to point it out. We have added more references to support the statement. Matuszak et al. showed the benefit of VMAT in pediatric cancer, bone marrow-sparing whole-abdominopelvic irradiation, lung and spine. Wu et al. 2 arc plans were comparable to IMRT for spine body radiotherapy.

**Line 64: "...various sites including prostate, spine, head and neck, etc.2,3" - Again, the references #2 and #3 cited are H&N and prostate only. There is no "spine". I believe there are references including abstracts on spine treated with RapiadArc available. The authors just need to more the relevant references to support their statement.**

We have added the reference from Kuijper about their clinical experiences about Spine SBRT to support the statement.

**Lines 64-65: "...Commissioning and QA of VMAT have been studied extensively 4-7": Reference #19 should be cited here as well because it's the first paper on the commissioning of Varian RapidArc.**

Included. Thank you.

**Lines 67-69: "However, no study has been presented previously to evaluate the planning and delivery accuracy of IMRT and RapidArc (the implementation of VMAT technique on TrueBeam system) treatments systematically during commissioning, to our knowledge" - This statement is not quite true.**

**There are publications on commissioning TrueBeam/FFF and IMRT & VMAT dose accuracy. I suggest the authors do a thorough literature search on this topic and rephrase their statements.**

Sorry for the incorrect statement. We did literature searches and included the reference “Verification of dosimetric accuracy on the TrueBeam STx: rounded leaf effect of the high definition MLC”. The authors have done a great job to evaluate systematically the dosimetric accuracy on the TrueBeam STx. The statement has been rephrased.

**Lines 138-139: "a hybrid approach was performed to optimized the setting of leaf transmission and DLG": Is the "hybrid approach" referring to "the combinations of DLG and transmission were optimized based on the ion chamber measurement results of TG-119 plans"? If yes, why was it based on ion chamber only? Why not film dosimetry? In my opinion, film dosimetry plays a more important role in the adjustment of DLG in beam modeling.**

We agree with the reviewer that the DLG and leaf transmission should be optimized based on both ion chamber reading and film dosimetry results. However, since the films had to be processed next day, in order to achieve the optimal value in a very tight commissioning schedule, we adjusted the values of DLG and transmission based on ion chamber reading, which was instantaneous, and then we validated the results from the films.

**Lines 178-182: This film dosimetry section deserves a little more detail; especially the EBT3 film is in the title of this paper. For example, how many calibration points? What is the maximum dose point in related to the IMRT/VMAT dose? Is one calibration curve for all IMRT/VMAT measurements? Are all calibration exposures on the same film using small fields or one large field for each dose point, etc.? In terms of scanning, were all calibration films scanned at the same time or one film at a time? All the technical details will be useful to the readers.**

Thank you for the recommendation. We have included the technical details of the film dosimetry.

Calibration films were irradiated for each photon energy with a nine 2x2 cm2 square dose pattern ranging from 0.4 to 3.2 Gy. For each energy, the calibration film was irradiated first and the corresponding plans were delivered under sequence for both IMRT and RapidArc. Since all the films were scanned 24 hours after delivery, the impact of time dependence on the dosimeric results was minimal. The sampled optical density values of each color channel were paired with the calculated dose values to establish the calibration curve through a cubic polynomial least squares fitting. The wait time from irradiation to scanning was approximately 24 hours for post-irradiation coloration. An Epson Expression 10000XL document flat-bed scanner (Seiko Epson Corp, Nagano, Japan) with Epson Scansoftware was used to scan the films. Each film was scanned in the center of the scanner bed to allow for better scanner response uniformity. The films were scanned in transmission mode for better scanning stability with settings of 75 dot per inch and 48 bit RGB mode (16 bits per color channel). Images were exported in tagged image file format (TIFF) for analysis and image processing filters were disabled.

**Line 281: "slice by slice" changes to "slice-by-slice".**

Corrected.

**Line 282: "three dimensional....2D": suggest changing to "3D...2D"**

**or define both "3D" and "2D" because they have never been defined previously in the text.**

We have included the definitions of 3D and 2D.

**General: The authors need to define IMRT, SBRT, SRS, EPID in the text (not only in abstract).**

Thanks to point out. We have included the definitions.

**General: PTW PinPoint ion chamber appears in the text at least 4 times. However, somewhere shows "pin point", somewhere shows "Pin Point". The authors should use a consistent format as the manufacturer stated, "PinPoint".**

Sorry for the inconsistency. We have corrected the format.

**General: The title of the paper said "commissioning ...using Gafchromic EBT3 films". However, the physics tools that the authors used for the commissioning included ion chamber (Tables 4-7), EPID (Figures 2-4), Gafchromic RTQA film (Figure 4). Overall, there are Figure 5 and Tables 8-9 showing EBT3 film results, as compared to many other figures and tables in this paper. Plus, there is lack of description on EBT3 film dosimetry method in this paper. Thus, the content does not seem to match the title, especially on "EBT3" part. I suggest the authors either change the title or add more details on EBT3 film IMRT/VMAT dosimetry.**

**Discussion: There is lack of discussion on EBT3 film dosimetry, except there are general statements regarding to EBT3. I understand that the authors stated, "the detail (EBT3 film) will be addressed in a separate paper" (line 334). But the title of this paper put a focus on EBT3 film and the readers expect more detail. For example, at least a basic question - Did the authors utilize single-color channel or multi-color channel dosimetry in this study? They have large differences in terms of accuracy and uncertainty between these 2 methods.**

Sorry for the misleading. We have changed the title to “IMRT and RapidArc Commissioning of TrueBeam Linear Accelerator”. Initially we included the film dosimetry component in the manuscript and realized that it could lead to a split objective for Linac commissioning and the utilization of the EBT films. Our integrated film dosimetry protocol was established to streamline a nine dose pattern delivery for dose calibration, calibration curve fitting, dose mapping from multiple color channels, and profile/gamma analysis. To quantify EBT3 film dosimetric uncertainties, films from eight packets were irradiated with open fields and standard deviations of dose maps were calculated at each color channel for evaluation. To determine the temporal dependence of EBT3 films, the average correction factors of different dose levels as a function of time were evaluated up to four days after irradiation.

We evaluated the dosimetric uncertainty of the optical density methods for three color channels over the dose range of 1.1 to 9.5 Gy. The mean uncertainties were 2.7%, 3.1% and 8.3% over 1~9Gy dose range for the red, green and blue channel respectively. Therefore the images from red and green channel were extracted and converted to dose using pre-established calibration curves. The dose maps were compared to the treatment planning dose matrix for subsequent profile and gamma analysis.

We have enriched the details of our film dosimetry protocol in the method by including the following statement.

Gafchromic film has excellent spatial resolution and is independent of beam angle, energy and dose rate. This makes it suitable for a variety of commissioning and treatment plan QA tasks, especially for FFF beams as dose rate is modulated for RapidArc delivery. We have developed an efficient film dosimetry protocol that converts optical density values to dose, establishes a calibration curve, registers film and planar dose and analyzes profile and gamma values. Calibration films were irradiated for each photon energy with a nine 2x2 cm2 square dose pattern ranging from 0.4 to 3.2 Gy. The sampled optical density values of each color channel were then paired with the calculated dose values to establish the calibration curve through a cubic polynomial least squares fitting. The wait time from irradiation to scanning was approximately 24 hours for post-irradiation coloration. An Epson Expression 10000XL document flat-bed scanner (Seiko Epson Corp, Nagano, Japan) with Epson Scansoftware was used to scan the films. Each film was scanned in the center of the scanner bed to allow for better scanner response uniformity. The films were scanned in transmission mode for better scanning stability with settings of 75 dot per inch and 48 bit RGB mode (16 bits per color channel). Images were exported in tagged image file format (TIFF) for analysis and image processing filters were disabled.

**Figure 3: The cause of the blue region in the center of all 3 picket-fence EPID images may not be totally due to "the large variation of signal through the radiation field for FFF beams" as the authors declared in the Discussion. Have the authors tried re-calibrating (dark/flood field) on the EPID before talking the FFF beam images? Also, the authors showed only the analysis on EPID images, not on the Gafchromic film. Is it because the authors have difficulty (software) to analyze the picket-fence tests from film? From my experience, at least 1000MU is needed for picket-fence test using GafChromic film and the signal was not very strong to be detected in any imaging analysis software. What MU did the authors use for their picket-fence tests using film? And what SSD of the film was placed for the test?**

Yes, we calibrated dark/flood field before delivering PF tests for all FF and FFF beams. Actually the blue regions shown in the figure is due to window leveling. The thresh hold was set 10.13 CU for FF and 4.89 CU for FFF. We have also included a figure to compare FF and FFF PF tests at the same gantry speed. The result was shown in Fig.4. The locations of the profile peaks were also within 1mm tolerance. Due to the difference of the energy spectrum between FF and FFF, the scatter contribution from FFF beam was less and therefore peak values were low instead. With the same window leveling, you can see that the blue region is more significant in the FF beams.

Actually we did not deliver PF tests on films since we were able to evaluate the images using EPID for FFF beams. The reason we used films for DR_GS and LS_DR tests for FFF beams is that portal dosimetry does not support FFF beams. Therefore we could not calculate dose deviation.

We agree with the reviewer that at least 1000 MU should be delivered to the films. We attached the films to the accessory mount and delivered 1500 MU to the films. The SSD was about 65cm.

**Figure 4: The images on both figures (b) and (d) don't look like EBT3 films as the figure legend states. They are more like GafChromic RTQA films.**

Sorry for the confusion of the figures. EBT3 is the only Gafchromic film we used in the commissioning. Figures (b) and (d) were cropped for better visualization purpose. I included the original image of figure (d) below for verification purpose.

**Also, both figures (a) and (c) have the same caption "with EPID for 6X". However, they are quite different. More explanations in the caption are needed to indicate their differences.**

Sorry for the confusion. We have rephrased the statement. Figure (a) and (b) are the test images to evaluate the accurate control of dose rate and gantry speed (DR_GS) during RapidArc delivery by delivering equal doses to seven 1.8 cm wide strips, with varying combinations of dose-rate, gantry range and gantry speed.

Figure (c) and (d) are the test images to evaluate the accurate control of the MLC leaf speed during RapidArc delivery by delivering 4 strips of equal dose but differing combinations of leaf speed and dose -rate (LS_DR).

**Table 1: The headers on the first row should be in consistent format - either use plural or singular.**

Sorry for that. It should be plural for all.

**Tables 6-9: Both table captions and contents (Table 6,7 vs. Tables 8,9) should be in a consistent format, such as "RapidArc" vs. "RA". The authors should either modify Tables 6, 7 or Tables 8,9 to make the same, either "RapidArc" or "RA".**

Corrected for “RapidArc”. Thank you.

*Reviewer B:*

**The paper describes IMRT and RapidArc commissioning using flattening-filter and flattening-filter-free modalities. Both types of treatment delivery and modality are investigated using the test plan cases described in AAPM TG-119, and the authors have established confidence limits as a baseline for patient specific treatment plan QA. This paper builds on the work of TG-119 by applying the test cases described in that report to flattening-filter-free modalities, and provides a useful reference for clinical physicists commissioning a Varian TrueBeam. It should also be of interest to physicists commissioning the new Elekta Versa. However, the paper suffers from a lack of detail in places and some poor English. I have included a list of my major and minor criticisms for the authors to consider.**

We really appreciate the reviewer to spend a lot of efforts to review the details and provide constructive feedbacks for us to improve the manuscript. We feel very sorry for the poor writing and will improve continuously.

Major comments

1. **Title. I think you should change the title of the paper and remove any reference to EBT3 films. The use of EBT3 film for commissioning is not really the focus of your paper, and you also state that the details of the film dosimetry will discussed in another paper. I would suggest changing the title to "IMRT and RapidArc Commissioning of a TrueBeam Linear Accelerator".**

Sorry for the misleading. We have changed the title to “IMRT and RapidArc Commissioning of TrueBeam Linear Accelerator”. Initially we included the film dosimetry component in the manuscript and realized that it could lead to a split objective for Linac commissioning and the utilization of the EBT films. Therefore we have removed the details of our film dosimetry program. Our integrated film dosimetry protocol was established to streamline a nine dose pattern delivery for dose calibration, calibration curve fitting, dose mapping from multiple color channels, and profile/gamma analysis. To quantify EBT3 film dosimetric uncertainties, films from eight packets were irradiated with open fields and standard deviations of dose maps were calculated at each color channel for evaluation. To determine the temporal dependence of EBT3 films, the average correction factors of different dose levels as a function of time were evaluated up to four days after irradiation.

2. **Lines 172-173. Provide some more details concerning your ion chamber calibration. This is critical to the reliability of your point dose measurements. Which calibration factor are you referring to? Did you apply a TG-51 formulism? Did you use another detector / calibration method to validate your calibration?**

We followed the recommendation from TG119 for ion chamber calibration. We have rephrased the statement to make it clear. The ion chamber calibration was performed by irradiating the phantom with 100 MU per field, using parallel-opposed 10 x 10 cm2 AP/PA fields arranged isocentrically. The conversion factor from ionization to dose was established using the ratio of the reading and the dose from the treatment planning system. The calibration factor was established by averaging the two conversion factors of the AP/PA fields. The purpose was to reduce the effects of daily output variations and differences of stopping power and mass energy absorption coefficient between solid water and liquid water.

We also used another method to validate the calibration factor. The output of the Truebeam was calibrated using an A12 chamber following TG 51 recommendation, then cross calibrated with a PinPoint chamber. The deviations between two calibration methods were around 2% due to the reasons mentioned above.

3. **Lines 178-182. Notwithstanding the fact that your film dosimetry protocol is going to be topic for another paper, I think you should include the details of how you calibrated your film in this paper. A brief overview of your scanning procedure would also be useful.**

Thanks for the comments. We have enriched the film dosimetry section in the method.

Gafchromic film has excellent spatial resolution and is independent of beam angle, energy and dose rate^1^. This makes it suitable for a variety of commissioning and treatment plan QA tasks, especially for FFF beams as dose rate is modulated for RapidArc delivery. We have developed an efficient film dosimetry protocol that converts optical density values to dose, establishes a calibration curve, registers film and planar dose and analyzes profile and gamma values. Calibration films were irradiated for each photon energy with a nine 2x2 cm2 square dose pattern ranging from 0.4 to 3.2 Gy. The sampled optical density values of each color channel were then paired with the calculated dose values to establish the calibration curve through a cubic polynomial least squares fitting. The wait time from irradiation to scanning was approximately 24 hours for post-irradiation coloration. An Epson Expression 10000XL document flat-bed scanner (Seiko Epson Corp, Nagano, Japan) with Epson Scansoftware was used to scan the films. Each film was scanned in the center of the scanner bed to allow for better scanner response uniformity. The films were scanned in transmission mode for better scanning stability with settings of 75 dot per inch and 48 bit RGB mode (16 bits per color channel). Images were exported in tagged image file format (TIFF) for analysis and image processing filters were disabled.

4. **Discussion. A large chunk of the Discussion section belongs in the Introduction and the Materials and Methods. Lines 255-294 (justification and background for this work) should be moved to the Introduction, lines 306-308 (explanation of EPID sensitivity) should be moved to the Methods, and lines. 323-334 (film info) should also be moved to the Methods. These changes would improve the clarity of your description of the measurements earlier in the paper. The Discussion should just be used for discussion of your results.**

Thanks for the suggestions. We have followed the suggestions to improve the clarify of our description. Lines 255-294 have been merged into the introduction. The EPID sensitivity has been moved to the method. The film dosimetry information has also been moved to the method.

Minor comments

**Please take the time to proof read your manuscript carefully. There are a lot of missing words and poorly constructed sentences.**

1.**Line 6. Change "The institutional confidence limits..." to "Institutional confidence limits...."**

Corrected.

2.**Line 8. Insert "the" immediately before "capability".**

Corrected.

3.**Line 9. Insert "an" immediately before "electronic".**

Corrected.

4.**Line 13. Change "attached in the dose calculation" to "included in the dose calculation".**

Corrected.

5.**Line 18. Change the first sentence to "The mean of the absolute dose variation for variable dose rate and gantry speed....".**

Corrected.

6.**Line 22-27. I'm assuming that these are the average 95% CLs obtained over the test cases (apart from the range values given in the parentheses).**

**You should explicitly state that the values are average 95% CLs.**

Yes, they are the average values. They have been corrected.

7.**Line 28-29. If you're not going to discuss your film protocol in the paper, I would change this statement. Your CLs are based on ion chamber as well as film measurements.**

We have rephrased the statement.

8.**Line 116-118. You need to explain what you used to measure the PF exposures somewhere around here.**

Corrected. We used EBT3 films for FFF mode and EPID for FF mode.

9.**Line 117. Change "Static PF tests were acquired" to "Static PF tests were performed".**

Corrected.

10.**Line 118. FWHM of what? Most clinical physicists will know what you're talking about here but for the sake of clarity you should be explicit.**

Thanks to point it out. We have rephrased the statement to “The full-width half maximum (FWHM) values were compared between profiles of leaf match lines”.

11.**Line 119. Change "The dynamic PF images delivered using RapidArc technique" to "Dynamic PF images acquired using a RapidArc technique".**

Corrected.

12.**Line 121. Can you give numerical examples of the intentional errors?**

The picket fence test with an intentional error of 0.5mm was performed.

13.**Line 122. What was the strip separation?**

The gap was 1.0 mm wide.

14.**Line 124-126. Consider putting these values into a table along with the values for the FFF beams.**

Thanks for the recommendation. We put these values to table 1 to make it easier to read.

15.**Line 129-130. How big was the open field? How many MU compared to each strip? Give a few more details here.**

Updated. The open field size was 14 x 40 cm^2^. 60 MU was delivered to the open field as well as each strip.

16.**Line 132-133. Which dose rates did you use for these leaf speeds? What is the maximum allowable leaf speed during RapidArc delivery at your institution?**

The corresponding dose rates were 960, 1440, 480 and 240 MU/min for FFF beams and 600, 600, 480, and 240 MU/min fir FF beams. The maximum allowable leaf speed at our institution is 2.5 cm/s.

**17. Line 138. Define DLG here. This is the first time this acronym is used outside the abstract.**

Corrected.

**18. Line 140. Insert the word "a" immediately before "CC13".**

Corrected.

**19. Line 142. How long did you run the beam for at each position?**

30 seconds. 200MU was delivered at each position with a constant dose rate of 400MU/min.

**20. Line 144. What was the leaf speed?**

The leaf speed was 0.73cm/s.

**21. Line 145. Some more information on the optimization would be useful here. Did you adjust the offset position of the leaves?**

We evaluated the MLC offset position before DLG measurement. The central line offset between two banks was within 0.1 mm as shown below.

The modeling inaccuracy has been noted to potentially be caused by the overlap between opposing leaves. In the Eclipse TPS, only a single DLG parameter is modelled for different field sizes and delivery techniques and it is likely to induce the modeling inaccuracy. Several papers have been published to discuss the optimization of MLC parameters (Kielar et al, Med. Phys. 39 (10), October 2012 and Chauvet et al., Phys. Med. Biol. 50 (2005)). We tuned the parameters of DLG and transmission in Eclipse so that the measurement results could agree with the calculated dose in Eclipse for all TG119 cases.

**22. Line 147. Change "portion" to "portions".**

Corrected.

**23. Line 152. Insert the word "a" immediately before "Lucy phantom".**

Corrected.

**24. Line 152-153. What do you mean by "out of the couch"?**

Sorry for the confusion. We have rephrased the statement “The phantom was irradiated with a PA field at three different locations, one where the phantom was extended over the couch so that there was no couch in the field, and at out of the couch, on the thin and thick portions of the couch”.

**25. Line 157. Change "dimension" to "dimensions" and change "the pin point chamber" to "a pin point chamber".**

Corrected.

**26. Line 159. Insert the word "the" immediately before "Anisotropic"**

**and "Eclipse".**

Corrected.

**27. Table 1. Consider having a separate column identifying the measurement location of the target / high dose region and a separate column for the avoidance structure / low dose region.**

Thanks for the recommendation. We have modified the table based on your recommendation.

**28. Line 187. Change "point dose measurement" to "point dose measurements".**

Corrected.

**29. Line 189. Change "1.96 is defined based on normal distribution that 95% of the data.." to "The factor 1.96 is derived from the normal distribution so that 95% of the data...".**

Corrected.

**30. Line 190. This statement doesn't make sense to me. How does a limit of 0.01 correspond to 99%? The multiplication factor (in this case 1.96) for your sigma value determines the % of events that fall within your limit.**

**Your confidence limits have been defined so that 95% of the data should fall within the limit. I wouldn't expect exactly 95% due to your small sample size, but I would expect something close.**

Thanks for your understanding that due to the small sample size from a single institution, it is an estimation of the confidence interval. As the reviewer mentioned, 1.96 is the multiplication factor for 95% probability that the interval contains the true population mean. Sorry for the incorrect statement of a limit of 0.001 corresponding to 99%. We have removed the statement.

**31. Line 208. Change "7" to "seven".**

Corrected.

**32. Tables 2 and 3. Insert the equation for DR_GS into the caption.**

Corrected.

**33. Lines 212-215. I think it's better to include the dose deviation values as part of a table rather than listing them in the text.**

The dose deviation values for variable DR_GS and LS_DR tests during RapidArc delivery are listed in Table 5.

**34. Line 215. Can you comment on how the output variation with angle affects your dose deviation results?**

Thanks for pointing it out. The output variation had contribution to the dose deviation. The overall deviation was a combination effect of dose rate, gantry angles, and output. However, the dose deviation results were much less than 1.70%. We revisited the measurements results and noticed that the maximum output variation was only 0.8%. The measurement results are included in the table below. We have corrected the data. Sorry for the mistake.

|  |  |  | **10x10** | | | | |
| --- | --- | --- | --- | --- | --- | --- | --- |
| **MU & Dose Rate** | **Angle** | **Data** | **6X** | **6X-FFF** | **10X** | **10X-FFF** | **15X** |
| **100 MU  400 MU/min** | 270 | **Average** | 0.171 | 0.166 | 0.188 | 0.184 | 0.196 |
|  |  | **Linearity** | **0.997** | **1.001** | **0.998** | **0.998** | **0.997** |
|  |  |  |  |  |  |  |  |
|  | 90 | **Average** | 0.170 | 0.165 | 0.188 | 0.183 | 0.196 |
|  |  | **Linearity** | **0.993** | **0.998** | **0.997** | **0.996** | **0.996** |
|  |  |  |  |  |  |  |  |
|  | 0 | **Average** | 0.171 | 0.165 | 0.188 | 0.184 | 0.196 |
|  |  | **Linearity** | **1.000** | **1.000** | **1.000** | **1.000** | **1.000** |
|  |  |  |  |  |  |  |  |
|  | 180 | **Average** | 0.172 | 0.166 | 0.189 | 0.185 | 0.197 |
|  |  | **Linearity** | **1.001** | **1.005** | **1.003** | **1.004** | **1.003** |

**35. Line 224. Change "0.9733 and 0.9846" to "0.9733 to 0.9846".**

Corrected.

**36. Line 225. Change "0.9794 and 0.9877" to "0.9794 to 0.9877".**

Corrected.

**37. Line 229 - 230. Change the first sentence to "Tables 6 and 7 show the ion chamber measurement results in the high-dose and low-dose regions, respectively, for both IMRT and RapidArc plans."**

Corrected.

**38. Line 231. Is the dose difference ratio the average dose difference ratio?**

Yes. The values presented are average value ± standard deviation.

**39. Line 233. Are the CL values outside the parentheses the average 95% CL values?**

Yes. The statement has been revised to make it clear.

**40. Table 6. Specify the definition of your % values in the caption.**

We have included the definition in the caption.

**41. Figure 5 caption. The blue line indicates the measured dose (not the green).**

Corrected. Sorry for the mistake.

**42. Line 242-243. Change the first sentence to "Tables 8 and 9 show the percentage of points passing the recommended 3%/3 mm Gamma criteria in the high-dose and low-dose regions, respectively, for both IMRT and RapidArc plans."**

Corrected.

**43. Line 304. Insert a period after "modes" and delete the rest of the sentence.**

Corrected.

**44. Line 314. Change "If measured values" to "If the unoptimized, measured values".**

Corrected.
