# Supplementary material for: IMRT and RapidArc commissioning of a TrueBeam linear accelerator using TG‐119 protocol cases
Source: J Appl Clin Med Phys. 2014 Sep 8;15(5):74–88. doi: 10.1120/jacmp.v15i5.4843 (PMC5711094; doi:10.1120/jacmp.v15i5.4843)
Supplement: Supplementary file 2 — Supplementary Material [file ACM2-15-074-s002.doc]

**Point-by-point responses**

Thank you to the associate editor and reviewer for their insightful comments on this work. Below we provide a point-by-point response to the reviewers’ questions and comments.

*Associate Editor Comments:*

General comments:

1. **The** **Please change the title of the paper to “IMRT and RapidArc**

**Commissioning of TrueBeam Linear Accelerator using TG-119 protocol cases” or an equivalent verbage.**

Thank you for this comment. We have changed the title of the paper to “IMRT and RapidArc Commissioning of a TrueBeam Linear Accelerator using TG-119 protocol cases”.

2. **Line 12. Please delete “all the cases were planned based on TG119 criteria.” This has already been said in the earlier sentence and is a repeat.**

Corrected.

3. **Line 25. Please add std dev to the mean values where ever you have used only average values along with range has been entered.**

Thank you for the suggestion. We have added standard deviation to the mean values. Additionally we found out an error in the calculation of the mean values. We have corrected this error throughout the manuscript. We apologize for the oversight.

4. **The wordings from line 145-164 have close semblance from the paper of Ling et al. It should be abridged and reworded.**

Thank you for pointing it out. This was not intentional. We have rephrased the writing as follows: (see lines 148-157).

A combination of 7 dose rates and gantry speed (DR_GS) as shown in table 1 were delivered to evaluate the control accuracy during RapidArc delivery. Equal dose was given to each of seven 1.8 cm wide strips using a different DR_GS. The deviation for each DR_GS combination was calculated by taking the ratio of the reading to the average value for all the positions.

 ( 2 )

Where, Rcorr(x) is the corrected reading at each strip, R(x) is a PF test image reading, Ropen(x) is an open field (14 cm2 x 40 cm2) image reading. 60MU was delivered to the open field as well as each strip. Similarly, accurate control of the MLC leaf speed was evaluated by delivering 4 strips of equal dose but differing combinations of leaf speed and dose rate (LS_DR) using the same methodology.

5. **In line 330, they have mentioned that IMRT & RapidArc pass rates of 3 & 7% respectively due to DLGs. This seems to be odd and should be investigated. Using a Mapcheck or equivalent device which also has attachment allowing it to hook up to the collimator. Thus the entire system will rotate along with the gantry in RapidArc. However, in relation to Gantry, it will be an IMRT delivery only i.e. rotational aspects will be neutralized. We have been using DLG without any adjustment and our results are fine.**

We completely agree with the associate editor that the discrepancy should be carefully investigated before drawing a conclusion. To ensure the delivery accuracy, we used three different devices (ion chamber, Matrixx and film) for the patient specific QA of the first 9 patients treated on the Truebeam machine using RapidArc techniques. We did not notice any systematic differences in the measurements between these devices.

| **Site** | **IC % diff** | **MatriXX 3/3** | **Film 3/3** |
| --- | --- | --- | --- |
| prostate | -1 | 99.7 | 99.2 |
| prostate | 1.7 | 93.24 | 99.4 |
| prostate | -1 | 100 | 99.85 |
| prostate | -1.9 | 98.06 | 99.1 |
| prostate boost | -1 | 95.5 | 100 |
| esophagus | -1.6 | 97.39 | 95.3 |
| prostate | -0.4 | 95 | 99.98 |
| esophagus, rect | -1.5 | 99.65 | 99.5 |
| esophagus boost | -0.3 | 99.2 | 95.7 |

During commissioning, we also compared the gamma results of four RapidArc plans measured with both EBT3 film and EPID for two energies. Very good agreement, as shown below, was noted between these measurement devices.

|  | 6X | | 18X | |
| --- | --- | --- | --- | --- |
|  | EBT3 | EPID | EBT3 | EPID |
| Prostate | 99.9 | 100.0 | 99.6 | 100.0 |
| MultiTarget | 99.8 | 100.0 | 99.6 | 99.9 |
| CShape hard | 99.9 | 100.0 | 100.0 | 100.0 |
| HN | 99.9 | 99.4 | 99.9 | 99.9 |

There are various methods to measure the DLG: (1) measuring the distance between the radiation and geometrical field edge of a MLC defined field size, (2) matching the gap width profiles with the measured values, (3) optimizing the parameters based on treatment delivery, (4) sweeping MLC leaves with a variety of sliding MLC gap widths. Each test has its pros and cons and associated uncertainties. Kielar et al. reported that the measured DLG value might not be suitable for VMAT as it could lead to as much as 5% discrepancies between the calculated and the measured dose. (Kielar KN, Mok E, Hsu A, Wang L, Luxton G. Verification of dosimetric accuracy on the TrueBeam STx: Rounded leaf effect of the high definition MLC. Med Phys. 2012;39(10):6360–71.) For the Eclipse TPS, only one DLG value can be commissioned for all different field sizes and delivery techniques. Therefore, there is a tradeoff in the optimal DLG between IMRT and VMAT-measured fields as well as the fields with different sizes and modulation.

6. **In 335, authors attribute the difference in DLG between different linacs to difference in the MLC leaf material/design and MLC calibration. This is a very strong statement without any scientific proof. The quality of these materials and workmanship is usually very high. Moreover, the MLC calibration technique has now been made electronic and does not have any subjective visual calibration as was common in old versions of MLC software’s.**

We agree with the reviewer and have rephrased the statement as follows at line 326:

The deviation could stem from various sources including measurement uncertainty, slight differences in MLC leaf material/design, etc. Therefore it is important to verify the DLG values by comparing the measured dose and calculated doses.

7. **In 336, authors say that the adjustments did not had any impact on the IMRT delivery. This needs more explanation. Was the IMRT field used for this comparison was less modulated e.g. prostate etc.**

The difference between the values of the DLG between IMRT and VMAT plans is caused by different contributions to the dose from the beam penumbra, which is a consequence of different patterns of leaf movement. Szpala et al. studied the sensitivity of dose calculation on the DLG values using a sliding window technique. They noticed that a single DLG value (2.0 mm) can be applied as the optimal value for all different widths of the MLC slits since the dose was integrated as the leafs travel across the chamber as a slit. The DLG values for smaller and larger regions were averaged out. We have added this statement in the text at line 338.

8. **Please limit the %difference in the tables to 1 significant digit only.**

Corrected.

9. **Table 10 & 11 please indicate the condition for gamma passing rate [e.g. 3%/3mm etc.]**

We have added 3%/3mm in the caption.

*Reviewer C:*

Major comments:

1. **Line 207-214. You mentioned in your response to my first set of comments that you used another method (TG-51) to validate the TG-119 chamber calibration. I think it would worthwhile to mention that here, especially since you report agreement to within 2%. It’s a reassuring second check on your ion chamber dose.**

Thank you for the recommendation. We have added the following text at line 209.

To validate the calibration factor, the PinPoint chamber was also cross calibrated with an A12 chamber following the recommendation of TG 51. The deviations between the two calibration methods were around 2% due to the reasons mentioned above.

2. **Line 220. Did you shoot your nine 2x2 cm2 square dose pattern on the same sheet of film? If so did you correct your doses for beam scatter?**

Yes, all nine square dose patterns were shot on the same sheet of film as shown below (a). A binary mask pattern (b) was applied to the registered film image for sampling. The sampled optical density values of each color channel were then paired with the calculated dose values (including the scatter dose) to establish the calibration curve. The reference doses were also verified with a pin point 31014 ion chamber. The point dose percent difference is shown in the table.

(a) (b)

(c) (d)

**Figure (a) A calibration film irradiated with a nine 2x2 cm2 square dose pattern ranging from 0.4 to 3.2 Gy; (b) A mask pattern used to sample optical density values to establish the calibration curve; (c) The corresponding planar isodose distribution; (d) film scanning orientation with the longer side of film aligned to the scanner detector array.**

| Point dose %difference | | |  |
| --- | --- | --- | --- |
|  |  | Gantry |  |
|  | -1.23% | 0.02% | 1.38% |
| Pt. Right | -1.14% | 0.13% | 0.29% |
|  | -1.22% | -1.15% | 0.05% |

3. **Line 223-224. The scanner lamp intensity can vary for the first few scans after the scanner is turned on (L. Paelinck et al, Phys Med. Biol. 52, 231 (2007)). Did you warm up the scanner lamp (i.e. by taking blank scans) before scanning your film?**

Thanks for pointing this out. The scanner was warmed up by 16 consecutive blank scans before each scanning session in our current practice. We have added this text and two references at line 223.

Ref 1. M. Martisikova, B. Ackermann and O. Jakel, "Analysis of uncertainties in Gafchromic EBT film dosimetry of photon beams," Phys Med Biol 53, 7013-7027 (2008).

Ref 2. L. Paelinck, W. De Neve, C. De Wagter, "Precautions and strategies in using a commercial flatbed scanner for radiochromic film dosimetry," Phys Med Biol 52, 231-242 (2007).

4. **Line 265 & Table 3. Does the data shown in Table 3 just correspond to one DR_GS or did you shoot each strip using a different DR_GS? You should clarify this in your Results and your Methods section.**

We apologize for the lack of clarity. Each strip was delivered with a different DR_GS. Table 1 lists the combinations of DR_GS used in the test: 176 MU/min at 4.8°/s, 352 MU/min at 4.8 °/s, 528 MU/min at 4.8°/s, 600 MU/min at 4.1 °/s, 600 MU/min at 3.3°/s, 600 MU/min at 2.9 °/s, and 600 MU/min at 2.6°/s for FF beams. Table 3 lists the dose deviation calculation at each of the seven strips. We have made changes in the methods and results to make this more clear.

Minor comments

**The following remarks are suggested changes that I think would improve the clarity of the language used in the paper.**

1. **Title. Insert the word ‘a’ before “TrueBeam”.**

Corrected. We have changed the title of the paper to “IMRT and RapidArc Commissioning of a TrueBeam Linear Accelerator using TG-119 protocol cases” per the recommendation of the associate editor.

2. **Line 28-29. Change the sentence to read “Based on ion chamber as well as film measurements, we have established CL values to ensure the high precision of IMRT and RapidArc delivery for both FF and FFF modalities.”**

Corrected.

3. **Lines 59-61. Insert a period after treatment delivery and delete the rest of the sentence.**

Corrected.

4. **Lines 63-64. Change the last sentence to “It is critical to develop QA protocols during commissioning to assure patient safety when using these new technologies.”**

Corrected.

5. **Line 71. Insert the word “an” before “impact”.**

Corrected.

6. **Line 76. Change “FFF beam has been introduced to increase in dose rate” to “The FFF modality has been introduced to increase dose rate”.**

Corrected.
